# Evaluation and Comparison of Treatment with Polymerised Collagen or Pirfenidone as Stand-Alone Therapy on the Production of Profibrotic Factors in Tracheal Stenosis and Tracheal Anastomosis Scarring After Resection

**DOI:** 10.3390/ijms27125332

**Published:** 2026-06-12

**Authors:** J. Raúl Olmos-Zuñiga, Mariana Silva-Martínez, José S. López-González, Miguel Gaxiola-Gaxiola, Avelina Sotres-Vega, Pablo Gomes-da Silva de Rosenzweig, Matilde Baltazares-Lipp, Lya Edith Pensado-Piedra, Fortunato Juárez-Hernández, Roberto Sotelo-Robledo, Laura Romero-Romero

**Affiliations:** 1 Experimental Lung Transplant Laboratory, Instituto Nacional de Enfermedades Respiratorias Ismael Cosio Villegas, Mexico City 14080, Mexico; raolzu@yahoo.com (J.R.O.-Z.); avesotve@yahoo.com (A.S.-V.); pablodasilvaros@gmail.com (P.G.-d.S.d.R.); 2 Pulmonary Cancer Laboratory, Instituto Nacional de Enfermedades Respiratorias, Ismael Cosio Villegas, Mexico City 14080, Mexico; slopezgonzalez@yahoo.com; 3 Morphology Laboratory, Instituto Nacional de Enfermedades Respiratorias Ismael Cosio Villegas, Mexico City 14080, Mexico; mogomig@yahoo.com; 4 Experimental Surgery Department, Instituto Nacional de Enfermedades Respiratorias Ismael Cosio Villegas, Mexico City 14080, Mexico; balmlipp@yahoo.com.mx; 5 Imaging Department, Instituto Nacional de Enfermedades Respiratorias Ismael Cosio Villegas, Mexico City 14080, Mexico; lyapensado@gmail.com (L.E.P.-P.); drjuarez.radiologo@gmail.com (F.J.-H.); dr.sorr@gmail.com (R.S.-R.); 6 Department of Pathology, Facultad de Medicina Veterinaria y Zootecnia, Universidad Nacional Autónoma de México, Mexico City 14080, Mexico; lromeror@unam.mx

**Keywords:** tracheal stenosis, restenosis, ITGβ1, TGF-β, fibronectin, elastin, collagen deposit, PFD, polymerised-collagen, MMC

## Abstract

Polymerised type I collagen (polymerised collagen) and pirfenidone (PFD) reduce fibrosis. However, their use as pharmacological monotherapy for tracheal stenosis (TS) has not been evaluated. This study aimed to evaluate the effects of polymerised collagen or PFD, used either as monotherapy for TS, or in combination with tracheal resection (TRE) on the development of restenosis (RTS), apoptotic body (AB) formation, production of profibrotic proteins, ITGβ1 expression and metalloproteinase (MMP) expression in a rat model of TS. Eighty Wistar rats underwent TS. Forty received monotherapy (treatment A) with: saline solution (SS), mitomycin C (MMC), polymerised collagen, or PFD. The other forty received TRE combined with the pharmacological treatment (treatment B). They were clinically evaluated. Four weeks after treatment, AB formation, ITGβ1, TGF-β, profibrotic proteins, and MMPs expression were assessed in TS tissue and post-TRE tissue. Our findings showed that treatment A with polymerised collagen or PFD did not reverse TS but halted its progression, reduced the expression of profibrotic proteins, ITGβ1 and MMP-9, and increased AB and MMP-1. Treatment B promoted normal tracheal healing and prevented RTS. We conclude that the use of polymerised collagen or PFD as monotherapy does not reverse TS. However, they halt disease progression by modulating the production of profibrotic factors. In combination with TRE, these agents promote favourable tracheal healing and prevent RTS.

## 1. Introduction

Benign tracheal stenosis (TS) is characterised by a narrowing of the tracheal lumen caused by fibrotic scar formation, which can be life-threatening due to the development of severe respiratory compromise [1,2]. TS is commonly caused by prolonged endotracheal intubation, tracheostomy, or external cervical trauma, leading to sustained inflammatory response of the tracheal mucosa. These processes promote the continuous production of inflammatory mediators and profibrotic cytokines in immune and stromal cells, dysregulating cellular differentiation and driving cell death. This ultimately leads to an imbalance between collagen synthesis and degradation, driven by excessive deposition of extracellular matrix (ECM) components. Mechanistically, these events lead to excessive granulation tissue proliferation and finally the development of TS [3,4].

The treatment of choice for TS is resection and end-to-end anastomosis of the affected tracheal segment (TRE). However, endoscopic management has increasingly been adopted as a less invasive alternative. Despite this, neither approach is fully effective, as previous studies have reported restenosis (RTS) rates ranging from 16% to 36.4% following surgical resection [5] and about 40% to 70% after performing endoscopic procedures [6]. This limitation is primarily due to recurrent injury to the tracheal mucosa, which predisposes to recurrent inflammation and may ultimately lead to pathological scar formation. For this reason, management of this condition is commonly combined with adjuvant pharmacological therapies, such as mitomycin C (MMC), corticosteroids, or antibiotics, aimed at preventing fibrosis development; however, their effectiveness has been variable [3,7]. Consequently, it is essential to investigate non-surgical therapeutic strategies that target and block the key molecular pathways involved in the fibrotic processes of TS and RTS [2] with the aim of reversing the established disease or halting its progression [3,7,8].

Previous studies have reported that polymerised type I collagen (polymerised collagen) [9] and pirfenidone (PFD) [10] prevent the development of TS during tracheal healing in animals undergoing TRE and in tracheotomised rats [7]. This effect is attributed to their role as wound-healing modulators (WHMs), which reduce inflammation and fibrosis. However, their efficacy as stand-alone treatments has not been evaluated once tracheal stenosis has already developed. Furthermore, there are no studies on the biological events that may be altered to reduce these pathological processes.

The objective of this study was to evaluate the effects of polymerised collagen or PFD, used either as monotherapy for TS, or in combination with tracheal resection (TRE) on the development of restenosis (RTS), apoptotic body (AB) formation, production of profibrotic proteins, integrin beta 1 (ITGβ1) expression and metalloproteinase (MMP) expression in a rat model of TS.

## 2. Results

Following the surgical procedures used to induce tracheal stenosis and perform tracheal resection, all animals developed mild stridor during the first postoperative week, which resolved by the second postoperative week. Although all animals survived the procedures used to induce tracheal stenosis and perform tracheal resection, not all survived until the end of the study period.

### 2.1. Clinical Findings

**Figure 1:**
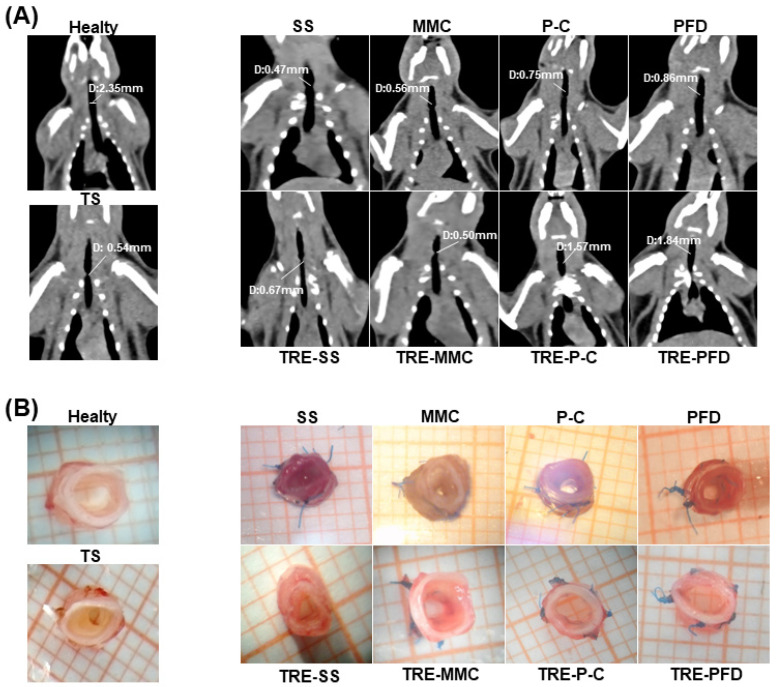
Tomographic and morphometric findings observed in TS at diagnosis and after treatment with SS, MMC, and WHM, alone or in combination with TRE. (**A**) Tomographic images demonstrating a reduction of more than 50% in tracheal lumen diameter at the time of TS diagnosis in animals that received pharmacological treatment alone and in those treated with SS and MMC combined with TRE; in contrast, animals treated with polymerised collagen (P-C) and PFD combined with TRE showed a reduction of less than 20%. (**B**) Morphometric images showing grade 2 TS at diagnosis and in animals treated only with P-C and PFD; grade 3 TS in animals treated with SS and MMC alone or in combination with TRE; and grade 1 TS in animals treated with P-C and PFD combined with TRE.

Tracheal stenosis was successfully produced in all animals in the fourth postoperative week, as confirmed by the presence of moderate stridor and dyspnea, as well as by a reduction in the diameter of the tracheal lumen observed on computed tomography (Figure 1A). Post-treatment, 100% of the animals in groups A and B treated with SS, subgroups I-A and I-B, and those treated with MMC, subgroups II-A and II-B (*p* = 0.001, Kruskal–Wallis and chi-square), required euthanasia during the first and third weeks after treatment due to the development of severe stridor and dyspnea at rest. In contrast, throughout the study period all animals in group A treated with polymerised collagen (subgroup III-A) or PFD (subgroup IV-A) exhibited moderate stridor, similar to that observed at the time of TS diagnosis, as well as moderate exertional dyspnea during the first two weeks post-treatment, which improved to mild dyspnea from the third week onward. Finally, 100% of the animals in subgroups III-B and IV-B, treated with surgery combined with WHMs presented only mild stridor during the first postoperative week, with an uneventful clinical course for the remainder of the study period (*p* = 0.001, Kruskal–Wallis). At the time of TS diagnosis, CT studies in all rats showed an average reduction in tracheal lumen diameter of 37% (1.39 ± 0.48 mm) compared with healthy rats (2.18 ± 0.15 mm) (*p* < 0.001, ANOVA, Dunnett). In addition, after pharmacological treatment, animals treated with SS or MMC showed greater progression of stenosis, with a reduction in tracheal lumen ranging from 61% (0.85 ± 0.26 mm) to 57% (0.93 ± 0.17 mm), respectively (*p* < 0.001, ANOVA, Dunnett, Tukey). In contrast, rats treated exclusively with polymerised collagen (39%; 1.34 ± 0.27 mm) or PFD (40%; 1.31 ± 0.15 mm) did not show significant progression of TS, as the tracheal lumen size remained similar to that observed at the time of diagnosis. However, animals treated with surgery combined with SS or MMC developed RTS, with tracheal lumen reductions of 37% (1.38 ± 0.41 mm) and 33% (1.45 ± 0.40 mm), respectively (*p* < 0.001, ANOVA, Dunnett, Tukey), compared with healthy trachea values and with groups III-B and IV-B. In contrast, animals treated with TRE combined with polymerised collagen (1.94 ± 0.21 mm) or TRE combined with PFD (1.93 ± 0.51 mm) showed a tracheal lumen reduction of less than 12% compared with baseline values prior to TS induction (2.18 ± 0.15 mm) (*p* < 0.001, ANOVA, Tukey). The reduction in tracheal lumen in these groups remained clinically insignificant.

### 2.2. Macroscopic and Morphometric Findings

In all cases (100%) in the subgroups that received pharmacological treatment alone and in those treated with TRE-SS or TRE-MMC who developed RTS, the anastomotic site appeared inflamed and hyperemic and exhibited moderate to severe fibrosis (*p* = 0.001, chi-square). Restenosis presented with an hourglass-shaped configuration. In contrast, in all animals treated with either polymerised collagen or PFD, in combination with TRE, the anastomosis showed adequate healing (Figure 1B). According to the morphometric analysis after TS production, a 46% reduction in tracheal lumen diameter (3.92 ± 0.86 mm) compared with healthy tracheas (7.30 ± 0.39 mm) (*p* < 0.005, ANOVA, Dunnett) was detected, corresponding to grade 2 TS. Following pharmacological treatment, animals receiving SS (3.12 ± 0.55 mm, corresponding to 57% tracheal obstruction [TO]) or MMC (3.39 ± 0.17 mm, 54% TO) progressed to grade 3 TS. In contrast, the polymerised collagen (4.18 ± 0.41 mm, 43% TO) and PFD (4.16 ± 0.58 mm, 43% TO) groups remained at grade 2 TS. Conversely, animals treated with TRE combined with SS (5.18 ± 0.58 mm, 30% TO) or MMC (5.35 ± 0.57 mm, 27% TO) (*p* < 0.005, ANOVA, Dunnett, Tukey) developed grade 2 RTS (*p* = 0.001, Kruskal–Wallis) compared with the diameter of healthy tracheal rings. In contrast, animals undergoing TRE in combination with polymerised collagen (6.85 ± 0.35 mm, 6% TO) or PFD (6.91 ± 0.29 mm, 5% TO) exhibited a reduction in tracheal lumen of less than 7%, corresponding to grade one TS.

### 2.3. Microscopic Findings

**Figure 2:**
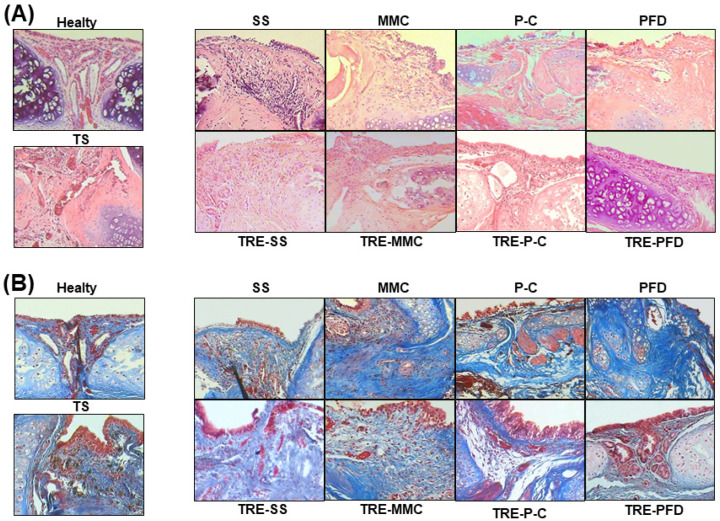
Representative micrographs of histological changes in tracheal tissue following administration of different treatments. (**A**) Degree of inflammation: severe in TS and in groups treated with SS and MMC, with or without TRE; moderate in animals treated with polymerised collagen (P-C) and PFD alone; and mild in those treated with P-C and PFD in combination with TRE. Hematoxylin–eosin staining (40×). (**B**) Degree of fibrosis: severe in TS and in groups treated with SS and MMC, with or without TRE; moderate in animals treated with P-C and PFD alone; and mild in those treated with P-C and PFD in combination with TRE. Masson’s trichrome staining (40×).

The histology of all healthy tracheal rings was normal. In contrast, stenotic rings without treatment, those treated with pharmacological therapy alone, and those that developed RTS exhibited epithelial metaplasia. Conversely, animals treated surgically in combination with polymerised collagen or PFD (*p* < 0.05, chi-square) showed areas of epithelial loss, partial re-epithelialisation, and focal epithelial hyperplasia. In addition, animals with TS and RTS treated with SS or MMC, with or without surgery, developed severe inflammation and fibrosis characterised by thick, disorganised collagen fibres (*p* < 0.001, Kruskal–Wallis). Contrasting with this, animals treated exclusively with polymerised collagen or PFD developed moderate inflammation and fibrosis with thick, disorganised collagen fibres. The subgroup treated with polymerised collagen in combination with TRE developed mild inflammation; whereas no inflammation was observed in those treated with PFD (*p* < 0.001, Kruskal–Wallis). Moreover, both groups exhibited mild fibrosis with thin, well-organised collagen fibres (*p* < 0.001, Kruskal–Wallis). In all subgroups, the inflammatory infiltrate consisted predominantly of lymphocytes; however, in animals treated with TRE combined with polymerised collagen or PFD, the lymphocytic infiltrate was mild (*p* < 0.001, Kruskal–Wallis) compared with the other study groups, in which it was moderate. Likewise, in all groups in which TS persisted and in those that developed RTS, a moderate number of blood vessels was observed. In contrast, in animals treated with polymerised collagen or PFD and TRE, as well as in healthy tracheas, the vascular proliferation was mild (*p* < 0.005, Kruskal–Wallis) (Figure 2).

### 2.4. Apoptotic Body Formation

**Figure 3:**
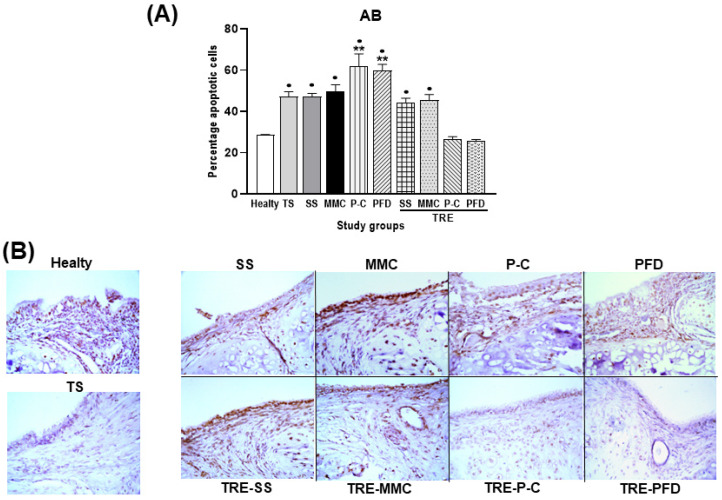
Presence of apoptotic bodies (ABs) in normal, stenotic, and treated tracheal tissue following pharmacological and surgical interventions. (**A**) Graph showing the percentage of apoptotic bodies in tracheal tissue across different groups. ● *p* < 0.008 ANOVA, Dunnett, Tukey; ** *p* < 0.003 ANOVA, Tukey. (**B**) Representative micrographs demonstrating an increased presence of apoptotic bodies (brown-stained cells) in tracheal tissue from pharmacologically treated groups, particularly TRE-SSF and TRE-MMC. Magnification 40×.

Apoptotic bodies (ABs) were observed in all animals across all tracheal layers, predominantly in the epithelium and tracheal mucosa. In healthy tracheas and in animals treated with polymerised collagen or PFD in combination with surgery, a similar level of AB expression was observed. In contrast, untreated stenotic rings and animals treated with SS or MMC (with or without surgery) showed moderate AB expression. By comparison, animals that received polymerised collagen or PFD monotherapy exhibited severe AB expression (*p* < 0.008, ANOVA, Dunnett, Tukey) compared with healthy tracheas and animals treated with polymerised collagen or PFD combined with TRE. Furthermore, the increase observed in animals from subgroups III-A and IV-A was significantly greater than that seen in animals treated with SS or MMC alone and in those that developed RTS (*p* < 0.003, ANOVA, Tukey) (Figure 3A,B).

### 2.5. ITGβ1 Protein Production Findings

**Figure 4:**
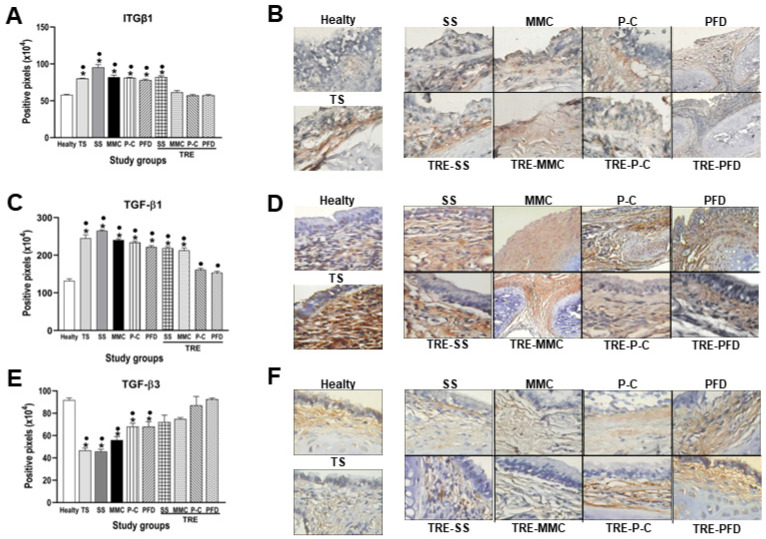
Expression of ITGβ1, TGF-β1, and TGF-β3 in tracheal tissue subjected to different treatments. (**A**) Graph showing ITGβ1 expression. ● *p* < 0.0001 ANOVA, Dunnett; * *p* < 0.0001 ANOVA, Tukey. (**B**) Representative micrographs demonstrating moderate immunostaining for ITGβ1 in TS and in groups treated with pharmacological therapy alone and SS combined with TRE; mild staining is observed in normal tissue and in groups treated with MMC, polymerised collagen (P-C), and PFD combined with TRE. Magnification 40×. (**C**) Graph showing TGF-β1 expression across the different study groups. ● *p* < 0.003 ANOVA, Dunnett; * *p* < 0.001 ANOVA, Dunnett, Tukey. (**D**) Representative micrographs showing strong immunostaining for TGF-β1 in TS, in all pharmacological treatment groups, and in TRE-SS and TRE-MMC groups. Magnification 40×. (**E**) Graph showing TGF-β3 expression in normal, stenotic, and treated tracheal tissue. ● *p* < 0.02 ANOVA, Dunnett; * *p* < 0.05 ANOVA, Dunnett, Tukey. (**F**) Representative micrographs demonstrating moderate immunostaining for TGF-β3 in normal tracheal tissue and in animals treated with P-C and PFD combined with TRE. Magnification 40×.

ITGβ1 expression was detected in all groups. Tracheal tissue from animals treated with TRE in combination with MMC, polymerised collagen, or PFD showed protein expression levels comparable to those observed in healthy tracheas. In contrast, stenotic rings prior to treatment and groups receiving pharmacological treatment alone exhibited a moderate increase in ITGβ1 expression. In contrast, animals that developed RTS and were treated with SS showed a marked increase compared with healthy tracheas and with groups treated with TRE combined with MMC or polymerised collagen, or PFD (*p* < 0.0001, ANOVA, Dunnett, Tukey) (Figure 4A,B).

### 2.6. Growth Factors Associated with Fibrosis Production Findings

#### 2.6.1. TGF-β1 Production

All subgroups showed increased TGF-β1 expression compared with healthy tracheas (*p* < 0.003, ANOVA, Dunnett). In untreated stenotic rings, in all subgroups that received pharmacological treatment alone, and in those that developed RTS, the increase was severe compared with healthy tracheas and moderate when compared with animals treated with surgery combined with polymerised collagen or PFD (*p* < 0.001, ANOVA, Dunnett, Tukey), which showed only a mild increase (Figure 4C,D).

#### 2.6.2. TGF-β2 Production

Although all experimental subgroups had increased TGFβ-2 expression, levels varied widely across the studied subgroups. Only tissue obtained from SS-treated animals without surgery showed statistical significance compared with healthy tracheas. (*p* = 0.026, ANOVA; *p* = 0.046, Dunnett).

#### 2.6.3. TGF-β3 Production

Regarding TGFβ-3 expression, only animals treated with TRE in combination with polymerised collagen or PFD showed values comparable to those observed in healthy tracheas. In the remaining groups, a reduction in TGFβ-3 production was observed, which was severe in untreated stenotic rings and in animals treated only with SS or MMC, and moderate in animals treated with polymerised collagen or PFD, all compared with healthy rings (*p* < 0.05, ANOVA, Dunnett). In all subgroups, after combined surgical and pharmacological treatment, TGFβ-3 production increased; however, only the increase observed in animals treated with surgery plus polymerised collagen or PFD was statistically significant compared with stenotic rings and animals receiving pharmacological treatment alone (*p* < 0.05, ANOVA, Tukey) (Figure 4E,F).

### 2.7. Fibronectin (FN) Production

**Figure 5:**
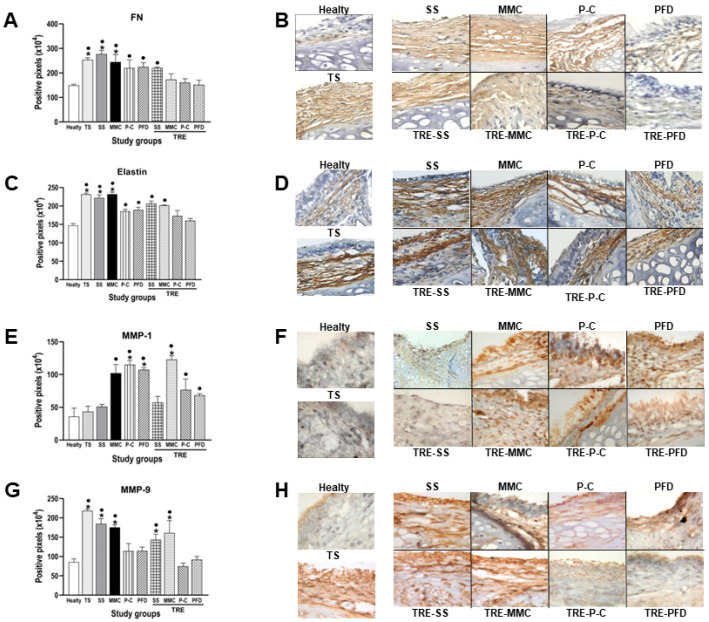
Expression of profibrotic proteins and ECM MMPs in tracheal tissue following administration of different pharmacological treatments. (**A**) Graph showing changes in fibronectin (FN) expression. ● *p* < 0.03 ANOVA, Dunnett; * *p* < 0.05 ANOVA, Tukey. Data are presented as mean ± SE. (**B**) Representative micrographs showing FN immunostaining: severe in TS and in groups treated with SS and MMC alone; moderate in groups treated with polymerised collagen (P-C) and PFD alone, as well as in SS and MMC combined with TRE; and mild in groups treated with P-C and PFD combined with TRE. Magnification 40×. (**C**) Graph showing elastin expression across the different study groups. ● *p* < 0.005 ANOVA, Dunnett; * *p* < 0.05 ANOVA, Tukey. Data are presented as mean ± SE. (**D**) Representative micrographs demonstrating strong elastin immunostaining in TS, in all pharmacological treatment groups, and in SS and MMC combined with TRE. Magnification 40×. (**E**) MMP-1 expression quantified by pixel intensity. ● *p* < 0.001 ANOVA, Dunnett; * *p* < 0.05 ANOVA, Tukey. Data are presented as mean ± SE. (**F**) Representative micrographs showing increased MMP-1 immunostaining in groups treated with P-C and PFD, with and without surgery, as well as in the TRE-MMC group. Magnification 40×. (**G**) Graph showing MMP-9 expression; ● *p* < 0.01 ANOVA, Dunnett; * *p* < 0.005 ANOVA, Tukey. (**H**) Representative micrographs demonstrating mild MMP-9 immunostaining in normal tracheal tissue and in tissue treated with P-C and PFD combined with TRE. Magnification 40×.

Immunohistochemical analysis showed increased FN expression in all study groups. This increase was severe in stenotic rings and in animals treated only with SS or MMC and moderate in those treated with polymerised collagen or PFD and in SS-treated animals that developed RTS, compared with healthy tracheas (*p* < 0.03, ANOVA, Dunnett). In contrast, animals treated with the combination of TRE and MMC, polymerised collagen, or PFD showed only a mild increase in FN expression, which was significant only when compared with groups exhibiting severe expression (*p* < 0.05, ANOVA, Tukey) (Figure 5A,B).

### 2.8. Elastin Production

Regarding elastin expression, stenotic rings and animals treated only with SS or MMC showed a severe increase, whereas animals treated with polymerised collagen or PFD and those treated with surgery combined with SS or MMC exhibited a moderate increase compared with healthy tracheas (*p* < 0.005, ANOVA, Dunnett). Animals treated with surgery in combination with polymerised collagen or PFD showed values similar to those observed in healthy tracheas. When comparing among groups, in stenotic rings obtained before treatment and in those treated with SS or MMC (with or without surgery), significant increases were found compared with those treated with surgery combined with polymerised collagen or PFD (*p* < 0.05, ANOVA, Tukey) (Figure 5C,D).

### 2.9. Biochemical Findings for Newly Formed Collagen (NFC)

Tissues from animals treated with TRE in combination with polymerised collagen (20,647 ± 3814) or PFD (17,644 ± 1645) showed NFC values (mg/g of tracheal tissue) similar to those obtained from healthy tracheas (18,433 ± 1581). In contrast, untreated stenotic rings (42,023 ± 1324), those treated with SS (without surgery: 45,393 ± 4181; with TRE: 44,340 ± 2431) or MMC (without surgery: 39,589 ± 6613; with TRE: 38,947 ± 2388) showed a severe increase in NFC, while animals treated only with polymerised collagen (29,707 ± 5856) or PFD (26,037 ± 793) exhibited a moderate increase. When groups were compared, both severe and moderate increases were significant compared with healthy tracheas (*p* < 0.0001, ANOVA, Dunnett). Nonetheless, only the severe increases were statistically significant when compared with animals treated with TRE in combination with polymerised collagen or PFD (*p* < 0.001, ANOVA, Tukey).

### 2.10. MMP-1 Production

In all groups, MMP-1 expression increased. This was mild in untreated stenotic tracheal rings and in SS-treated animals with or without surgery. In animals treated only with MMC, either alone or in combination with surgery, as well as in those treated only with polymerised collagen or PFD, the increase was severe. Conversely, in animals treated with polymerised collagen or PFD in combination with TRE, the increase was moderate compared to healthy tracheas (*p* < 0.001, ANOVA, Dunnett). When comparing between groups, only the severe expression showed by subgroups IIA, IIIA and IVA was statistically significant versus healthy tracheas, stenotic tracheal rings from untreated animals, those treated with SS (as a single treatment and in combination with TRE), and from animals treated with surgery combined with polymerised collagen or PFD (*p* < 0.05, ANOVA, Tukey) (Figure 5E,F).

### 2.11. MMP-9 Production

In all groups, increased MMP-9 expression was observed. This increase was severe in untreated stenotic rings and in animals treated only with SS or MMC, and moderate in those treated with TRE combined with SS or MMC, compared with healthy tracheas (*p* < 0.01, ANOVA, Dunnett). In animals treated only with polymerised collagen or PFD, the increase was mild compared with stenotic rings; however, this did not reach statistical significance. In contrast, animals treated with polymerised collagen or PFD in combination with surgery showed MMP-9 expression levels similar to those of healthy tracheas, which was statistically significant when compared with animals treated with SS or MMC with or without surgery (*p* < 0.005, ANOVA, Tukey) (Figure 5G,H).

## 3. Discussion

The treatment of choice for TS is TRE; however, it is not effective in all cases. Consequently, to prevent fibrosis and RTS, TRE is combined with drugs such as MMC. Nevertheless, these strategies have not been fully successful because they do not act on all phases of wound healing [3,7]. This underscores the need to identify WHM that interfere with the molecular pathways involved in tracheal fibrosis, prevent TS and RTS, reverse established disease, or block its progression. Therefore, the objective of this study was to evaluate the usefulness of polymerised collagen or PFD as monotherapy for TS and in combination with TRE on the development of restenosis (RTS), AB formation, expression of profibrotic proteins, and metalloproteinases in rats with TS.

In this study, we observed that, when used as monotherapy in established TS, these WHMs do not reverse stenosis but halt its progression by reducing the production of profibrotic factors. We also confirm that when these agents are applied as adjuvants, they prevent secondary surgical damage and inhibit the development of RTS by modulating the expression of factors that promote fibrosis.

The progression to grade 3 TS and the development of RTS observed in the subgroups treated with SS or MMC, either alone or in combination with surgery, were due to neither agent effectively preventing inflammation or the mechanisms responsible for fibrosis [11]. Likewise, our results are consistent with findings from other studies investigating the molecular mechanisms of TS, which reported that the use of MMC as an adjuvant for treating this condition offers limited clinical benefit and is unlikely to alter the natural course of the disease [2,12]. Nevertheless, our findings differ from those of other authors who have evaluated various pharmacological agents for TS and reported that MMC is effective in acute lesions by inhibiting the proliferative phase of wound healing [7,11], a finding that was not observed in the present study, as MMC did not prevent RTS.

Regarding the use of polymerised collagen or PFD as monotherapy for TS, there are no previous reports. However, the stabilisation of the progression of clinical, tomographic, and morphometric of TS observed in animals treated exclusively with these agents suggests a mechanism similar to that reported in patients with idiopathic pulmonary fibrosis and hypertrophic skin scars, where PFD and polymerised collagen halt the fibrotic process by blocking inflammation but without complete reversal of established fibrosis [13,14]. This suggests that using these agents as monotherapy may reduce clinical alterations in patients with TS or RTS who are initially unable to undergo invasive procedures, as described by Li et al. [6]. In subgroups treated with TRE combined with these WHMs, the findings demonstrated that these WHMs can prevent RTS when applied during the early stages of the fibrotic process, which is consistent with reported findings in other studies of tracheal healing [7,9,10].

Microscopically, the epithelial metaplasia observed in animals with persistent TS and RTS is a feature seen in patients with prolonged intubation or tracheostomy, in whom the epithelium heals as stratified squamous epithelium rather than ciliated respiratory epithelium. This shift is associated with chronic inflammation and apoptosis of ciliated cells [1]. As opposed to this, the epithelial hyperplasia observed in animals treated with TRE combined with polymerised collagen or PFD is a distinctive result of tissue remodelling in the upper respiratory tract, characterised by an increased number of cells of the same type and is a reversible process associated with inflammation [15].

The severe inflammation and fibrosis observed macroscopically and microscopically in animals treated with SS and MMC (with or without surgery) provoked a profibrotic response and accumulation of NFC [12], in the form of thick, disorganised collagen fibres, as described by Otteson et al. [16], when studying the temporal sequence of wound healing in the airway. In contrast, the well-organised collagen fibres observed in animals treated with TRE and polymerised collagen were probably due to the fact that during normal healing, there is a greater expression of decorin, which creates spacing between collagen fibrils, prevents their fusion, and ultimately limits fibre thickening [9]. In animals treated with PFD, this effect could be attributed to its regulation of collagen synthesis and processing, resulting in thinner collagen fibrils [17]. Similarly, in animals with TS treated exclusively with these WHMs, collagen fibre disorganisation and increased thickness were likely attributable to persistent moderate inflammation, which delayed the remodelling phase, and/or to an insufficient duration of treatment [9].

The moderate lymphocytic infiltrate observed in groups with TS and RTS was due to the fact that in this pathology, inflammatory infiltrates are mainly mediated by T lymphocytes [2], which secrete inactive TGF-β [18], while the reduced presence of lymphocytes in the other subgroups was attributed to the fact that polymerised collagen [19] and PFD [20] decrease their expression. Likewise, the moderate vascularisation and macroscopic hypermedia observed in animals with persistent TS and RTS may have occurred because tracheal scarring was still in the proliferative phase of wound healing, during which a new capillary network develops to restore perfusion. In contrast, treatment with polymerised collagen or PFD combined with surgery accelerated the remodelling phase, leading to a nearly avascular and acellular scar [1,2,12].

Apoptotic bodies were observed in all animals, as apoptosis during wound healing regulates the clearance of inflammatory, epithelial, endothelial, and fibroblast cells that have completed their function and facilitates ECM reorganisation; however, dysregulation of this process predisposes to pathological scar formation [21]. In animals treated with SS or MMC, in whom TS persisted, or RTS developed, fewer ABs were observed because, after tracheal injury, repeated inflammatory reactions promoted the release of inflammatory factors and profibrotic cytokines (such as TGF-β1), which inhibit apoptosis and predispose to greater ECM deposition and excessive granulation tissue proliferation, ultimately leading to TS [3]. However, our findings in the group treated with TRE combined with MMC do not agree with those reported by Ekinci et al. [11], who described that topical MMC induces fibroblast apoptosis when used prophylactically for TS. Similarly, animals treated exclusively with polymerised collagen or PFD in whom TS persisted exhibited a marked increase in ABs. This is because polymerised collagen modulates the expression of leukocyte-associated immunoglobulin-like receptors (LAIR-1), activates M1 macrophages, and regulates inflammatory mediators such as CXCL1, CXCL10, CCL2, as well as macrophage inflammatory proteins. Together, these effects enhance apoptosis, attenuate the inflammatory response, and support appropriate tissue repair [19], while PFD blocks the expression of profibrogenic growth factors and survival factors [22]. On the other hand, the similar levels of ABs to those of healthy tracheas in animals treated with TRE combined with polymerised collagen or PFD were due to the fact that, after removal of damaged tissue and the preventive administration of these drugs, wound healing was accelerated and most fibroblasts, immune cells, and endothelial cells had already undergone apoptosis [12]. In normal wound healing, this effect begins around day 12, peaks at approximately 20 days, and disappears by day 60 [21].

Regarding ITGβ1, its presence across all groups was observed because it associates with certain alpha chain integrins, forming heterodimeric cell surface receptors for collagen and acting as a mechanical link between the cell and the ECM, participating in the initiation of signal transduction pathways involved in multiple physiological and pathological cellular functions (such as activation of latent TGF-β) [8,23,24]. The increased expression (moderate to severe) of this integrin in stenotic rings, both prior to treatment and following pharmacological interventions, was likely driven by the heightened inflammatory response in these animals, which promoted its upregulation [25]. These findings also suggest that, once TS was established, polymerised collagen or PFD only partially reduced integrin expression, perhaps because active fibroblasts were still capable of producing it [26]. The similar expression observed in the tracheas of animals treated with surgery combined with polymerised collagen or PFD, and in healthy tracheas, has not been previously reported. However, this may have been due to both agents reducing inflammation and inhibiting fibroblast activation and proliferation [8,19,27]. Alternatively, the anti-inflammatory effect of these drugs could inhibit the expression of deubiquitinating enzymes, favouring the degradation of the β subunit of integrins and its subsequent detachment from the membrane. This would have limited myofibroblast differentiation and excessive ECM deposition, as described in studies on ubiquitination pathways that regulate ITGβ1 and β5 levels in fibrotic tissues [28].

The increased expression of TGF-β1 and TGF-β2 in TS and RTS could be caused by the fact that these animals presented a greater lymphocytic infiltrate, expression of MMP9 and a lower amount of AB [6,18,29] that favours their production. In addition, high infiltration could contribute to increased ITGβ1 production, which activates latent TGF-β1 stored in the ECM, generating a positive feedback loop and further increasing its expression [28]. Regarding TGF-β2, no previous studies have reported its presence in TS. However, our findings suggest an increase during TS to recruit fibroblasts to the wound-healing site, as observed in pathological skin scars [30]. Nevertheless, in both TS and RTS, it may not play a major role in disease development, since the increase was not significant and was observed in both the pharmacological treatment alone and the combined surgical treatment groups. The reduced expression of TGF-β3 observed in stenotic rings after pharmacological treatment and in restenosis may be explained by its downregulation during the proliferative and remodelling phases of wound healing, stages in which TGF-β1 concentrations increase following epithelialisation [31]. Additionally, because TGF-β1 is the predominant isoform expressed during fibrotic responses, competitive binding among TGF-β isoforms to transmembrane receptors (TβRs) may further suppress TGF-β3 signalling [32]. Animals treated with TRE combined with polymerised collagen or PFD showed lower expression of TGF-β1 and TGF-β2 because both drugs reduce inflammation and block their production [2,7,14,27,33]. Furthermore, the combination of these drugs with resection of damaged tissue and approximation of healthy tissue allowed for normal wound healing, during which the expression of these factors and their receptors decreased [7,10]. In PFD-treated groups, this reduction may also be explained by the drug’s mechanism of action, which inhibits furin expression, an enzyme involved in TGF-β maturation [34]. Animals treated with TRE combined with polymerised collagen or PFD showed TGF-β3 levels similar to those of healthy tracheas and did not develop RTS, likely because preventive administration after removal of stenotic tissue inhibited inflammation and regulated its expression, thereby promoting their antifibrotic effects [9,10]. On the other hand, it is worth mentioning that the findings in animals treated with polymerised collagen or PFD alone suggest that once TS is established, these drugs do not completely prevent TGF-β1 expression; however, they reduce it, possibly by decreasing inflammation and ITGβ1 expression, which may explain why ECM protein production did not increase and TS did not progress.

The increased expression of FN, elastin, and NFC in stenotic rings before and after pharmacological treatment, as well as in RTS subgroups, is explained by the fact that this pathology results from hypertrophic scar formation, characterised by elevated levels of these ECM components [1,35,36]. This may also be due to higher TGF-β1 expression and reduced apoptosis, promoting fibroblast-to-myofibroblast differentiation and continued ECM protein production, leading to fibrosis [8,12,36]. Additionally, elevated ITGβ1 levels may have favoured their incorporation into the ECM [37]. In contrast, animals treated surgically in combination with PFD showed lower expression of these ECM proteins, as PFD has been reported to suppress the expression of type I collagen, FN, and elastin mRNA by inhibiting TGF-β1 production and signalling [20,38]. Although there are no studies evaluating the direct effect of polymerised collagen on FN and elastin expression, the data obtained in our study support the idea that a decrease in TGF-β production reduces the synthesis of collagen and ECM proteins. In animals treated with pharmacological therapy alone or those developing RTS, ECM component persistence may reflect established fibrosis. However, lower levels observed with polymerised collagen or PFD confirm reports in skin [14] and idiopathic pulmonary fibrosis [13] showing delayed fibrosis, suggesting a similar effect in TS by delaying the proliferative phase of wound healing. Findings in MMC-treated groups are consistent with reports indicating that MMC inhibits the production of several ECM proteins during the remodelling phase, although it has limited effects on collagen deposition [11].

All groups showed MMP-1 and MMP-9 expression, as these enzymes participate in all phases of wound healing by degrading damaged ECM proteins and cells, and by activating growth factors and cytokines [39]. Low MMP-1 levels in untreated stenotic rings and RTS groups may reflect hypertrophic scar formation, where persistent inflammation increases TGF-β1 and TIMP-1 levels, inhibiting MMP-1 expression and favouring excessive scarring [35,40]. Moreover, although MMP-1 expression occurs rapidly after injury, it decreases during the proliferative phase [35].

Severe MMP-1 expression in MMC-treated animals (with or without surgery) may be due to increased mRNA expression induced by this drug, as described by Seet et al. [41]. However, the specific pathway remains unclear. Similarly, severe MMP-1 expression in animals treated only with polymerised collagen or PFD, and moderate expression in those treated surgically with these WHMs, may be explained by inhibition of TGF-β1 by both agents [4,9,42,43], which promotes MMP-1 expression. In animals treated with polymerised collagen or PFD alone, the higher MMP-1 activity may reflect its role during the remodelling phase of wound healing [39]. In contrast, in the combined surgical treatment groups, MMP-1 expression was lower, consistent with its downregulation after wound closure and tissue repair [44].

The severe expression of MMP-9 observed in untreated stenotic rings, in animals treated with SS or MMC, and in those receiving these agents combined with TRE may be explained by its role in degrading gelatin generated during ECM collagen breakdown [35], a hallmark of both clinical and experimental TS [1]. In addition, MMP-9 is also expressed during the inflammatory and proliferative phases of wound healing, where it facilitates cell migration, suppresses integrin expression [45], and promotes the activation of TGF-β1, TGF-β2, and VEGF [2,35]. In PFD-treated animals, moderate expression after monotherapy and mild expression after combined treatment may be due to reduced synthesis and secretion of TGF-β1, as observed in idiopathic pulmonary fibrosis and cardiac and renal fibrosis models [20]. Although no studies describe the effect of polymerised collagen on MMP-9 expression, inhibition of inflammation and TGF-β1 production likely contributed to its reduced expression [9,14,27,42].

Although our study allowed us to advance our knowledge of the biological effect of two drugs on TS and during tracheal restenosis, it has limitations, such as the short duration of the proposed model, which does not allow us to evaluate whether drug therapy alone can reverse TS, as described in skin studies showing the regression of hypertrophic scars treated with polymerised collagen [14]. Additionally, we believe that TS models should be developed in larger animals to allow endoscopic evaluation and treatment, thereby assessing the effects of these newly implemented WHMs in combination with endoscopic interventions.

## 4. Materials and Methods

### 4.1. Experimental Animals

A randomised, prospective, longitudinal study was conducted using 80 clinically healthy Wistar rats (strain Hsd:WI), both sexes, aged 8–11 weeks and weighing 250–350 g, obtained from the vivarium of the Instituto Nacional de Enfermedades Respiratorias Ismael Cosío Villegas (INER). The study protocol was approved by the INER Bioethics Committee (protocol number B02-17) and carried out in accordance with the Mexican Official Standard for the Care and Use of Laboratory Animals (NOM-062-ZOO-1999) [46] and the U.S. Guide for the Care and Use of Laboratory Animals [47]. All methods were carried out in accordance with the ARRIVE guidelines and regulations. All procedures were performed under general anaesthesia, and every effort was made to minimise animal suffering.

### 4.2. Experimental Design and Study Groups

TS was produced in all animals by resection of four tracheal rings, which were immersed in bleomycin; subsequently, three rings were re-anastomosed in their original position to simulate the irritation, ischemic injury, and mucosal necrosis produced by an endotracheal tube, as described by Silva-Martínez et al. [40]. Once TS was established, forty animals were included as Group A, divided into subgroups of 10 rats each, and treated as follows:

**Subgroup I-A (n = 10):** TS treated with physiological saline solution administered subcutaneously (TS-SS).

**Subgroup II-A (n = 10):** TS treated with extraluminal topical application of mitomycin C (1.2 mg/kg) (TS-MMC).

**Subgroup III-A (n = 10):** TS treated with polymerised collagen (2.5 mg/kg) administered subcutaneously for 4 weeks (TS–polymerised collagen).

**Subgroup IV-A (n = 10):** TS treated with PFD (40 mg/kg/day) administered orally for 4 weeks (TS-PFD).

The remaining forty animals (Group B) underwent surgical treatment with TRE combined with pharmacological therapy according to their assigned study subgroup:

**Subgroup I-B (n = 10):** TS treated with TRE and physiological SS administered each week subcutaneously during 4 weeks (TRE-SS).

**Subgroup II-B (n = 10):** TS treated with TRE and extraluminal topical application of mitomycin C (1.2 mg/kg) (TRE-MMC).

**Subgroup III-B (n = 10):** TS treated with TRE and polymerised collagen (2.5 mg/kg) administered subcutaneously every week for 4 weeks (TRE-polymerised collagen).

**Subgroup IV-B (n = 10):** TS treated with TRE and PFD (40 mg/kg/day) administered orally for 4 weeks (TRE-PFD).

### 4.3. Anaesthesia and Surgical Procedure

For both TS production and TRE, all animals were anaesthetised with ketamine (Anesket^®^, Pisa, Guadalajara, Mexico) at 120 mg/kg intraperitoneally and xylazine (Rompun^®^, Bayer, Leverkusen, Germany) at 5 mg/kg intraperitoneally. A longitudinal incision was made in the midline of the ventral cervical region to expose the trachea, which was dissected circumferentially. The tracheal rings affected by TS were resected, including one healthy proximal ring and one healthy distal ring. Subsequently, a TRE was performed using non-absorbable polypropylene suture (Prolene^®^, Ethicon, NJ, USA) 7-0, with a continuous suture in the membranous portion of the trachea and interrupted sutures in the cartilaginous part.

After completion of the anastomosis, the incision was closed in a conventional manner. All animals received flunixin meglumine (Napzin^®^, Pisa, Mexico City, Mexico) at 4 mg/kg subcutaneously for postoperative analgesia and enrofloxacin (Baytril^®^, Bayer, Leverkusen, Germany) at 5 mg/kg subcutaneously for antibiotic prophylaxis. In addition, during the first three postoperative days, animals underwent nebulisation with salbutamol (Ventolin^®^, GlaxoSmithKline, Durham, UK) at a dose of 0.15 mg/kg diluted in physiological saline to a final volume of 4 mL, delivered with an oxygen flow of 5 L/min to prevent postoperative secretion accumulation [40].

### 4.4. Treatment

In subgroups I-A and I-B, 0.5 mL of physiological saline solution (SS) was administered subcutaneously every week for 4 weeks. In the MMC (Mixandex^®^, Pisa, S.A. de C.V., Guadalajara, Mexico) subgroups (II-A and II-B), 1.2 mg/kg of the drug (the dose used for TS treatment) was applied topically for five minutes; thereafter, the trachea was irrigated with SS [10]. The dose of polymerised collagen (Fibroquel^®^, Aspid S.A. de C.V., Mexico City, Mexico) used in subgroups III-A and III-B corresponded to that employed in experimental studies of tracheal wound healing (2.5 mg/kg) [10,48]. Animals treated with PFD (KitosCell^®^, Cell Pharma, S. de R.L. de C.V., Mexico City, Mexico) in groups IV-A and IV-B received the dose of 40 mg/kg/day, which has been shown to modulate tracheal wound healing in experimental models [10].

### 4.5. Evaluations

The study duration was 8 weeks. During the first 4 weeks, TS was produced, and during the remaining 4 weeks, the animals received treatment according to their study group. Throughout the study period, clinical and computed tomography evaluations were performed. At the end of the study, the TS site or the tracheal anastomosis was assessed macroscopically, morphometrically, and microscopically. Finally, we evaluated AB formation, the expression of the three TGF-β isoforms, ITGβ1, quantified NFC, and the expression of fibronectin, elastin, MMP-1, and MMP-9.

Clinical evaluation: This evaluation was performed daily during the first postoperative week and every third day thereafter, with particular attention to stridor and dyspnea.

### 4.6. Computed Tomography Evaluation

All rats underwent computed tomography (CT) with axial and coronal cervical images to measure tracheal lumen size. Scans were performed before TS induction, weekly until TS development, and weekly after treatment initiation.

### 4.7. Macroscopic Evaluation of the Trachea with TS and Post-Treatment

At the end of the study period, all animals were euthanised with an overdose of sodium pentobarbital (150 mg/kg/IP) (Anestesal, Pfizer S.A. de C.V., Guadalajara, Mexico). The stenotic tracheal segment was excised in animals receiving pharmacological treatment alone, and the anastomotic segment was removed in those receiving combined treatment. Scar characteristics were evaluated macroscopically. Tracheal lumen circumference was assessed by morphometry, and the degree of TS or RTS after treatment was determined according to the scale described by Freitag et al. [49]. For this purpose, the tracheal lumen circumference of healthy rings removed prior to TS production (baseline) at the time of TRE and at the end of the study was compared. The resected segment was then opened along the membranous portion to evaluate mucosal healing. A portion of the membranous wall was immediately frozen at −70 °C for biochemical quantification of the newly formed collagen (NFC) in the TS and tracheal anastomosis scar. The remaining tissue was used for histological and immunohistochemical analysis.

### 4.8. Microscopic Evaluation of the TS and Tracheal Anastomosis Scar

Samples obtained from the TS zone (stenotic rings) and the anastomosis site were fixed in 10% buffered formalin and embedded in paraffin. Sections of 4 µm were cut and slides were stained with hematoxylin–eosin and Masson’s trichrome. The samples were independently reviewed by two pathologists with extensive experience in lung disease, who assessed the degree of inflammation, fibrosis, and collagen fibre morphology, as well as the distribution of each parameter across the entire circumference of the sample, using a subjective scale based on severity [10,48].

### 4.9. Quantification of the Percentage of Apoptotic Cells

Cell death was determined by enzymatic labelling of free 3′-OH ends of fragmented DNA in apoptotic cells using the TUNEL method (TdT-mediated dUTP nick end labelling). After tissue fixation and mounting of slides as described for immunohistochemistry, the assay was carried out according to the manufacturer’s instructions (ApopTag Plus Peroxidase In Situ Apoptosis Detection Kit S7101, EMD Millipore Corporation, Carlsbad, CA, USA). The number of apoptotic cells was calculated using the apoptosis index (AI): AI = (apoptotic cells/total cells) × 100%. Sections were analysed in 10 random fields at 40× magnification.

### 4.10. Quantification of TGF-β Isoforms, ITGβ1, FN, Elastin, MMP-1, and MMP-9 Expression

At the TS site and tracheal anastomosis scar, protein expression of the three TGF-β isoforms, integrin β-1, fibronectin, elastin, MMP-1, and MMP-9 was assessed by immunohistochemistry (IHC). The following rabbit polyclonal antibodies against TGF-β1 (1:200 dilution, Abcam, Cat. No. ab25121, Cambridge, UK), TGF-β2 (1:50 dilution, Abcam, Cat. No. ab53778, Cambridge, MA, USA), TGF-β3 (1:100 dilution, Abcam, Cat. No. ab15537, Cambridge, UK), integrin β-1 (1:500 dilution, Boster, Cat. No. M00772-1, Pleasanton, CA, USA), fibronectin (1:250 dilution, Abcam, Cat. No. ab2413, Cambridge, UK), elastin (1:50 dilution, Abcam, Cat. No. ab21610, Cambridge, UK), MMP-1 (1:250 dilution, Abcam, Cat. No. ab38929, Waltham city, MA, USA), and a mouse monoclonal antibody against MMP-9 (1:200 dilution, Lifespan Biosciences, Cat. No. LS-B2182, Seattle, WA, USA) were used. The detection of primary antibodies was performed using the biotin-streptavidin-peroxidase system, with the ABC-HRP VECTASTAIN Rabbit IgG kits (PK-6101, Vectastain, Vector Laboratories, Newark, CA, USA) and ABC-HRP VECTASTAIN Mouse IgG kits (PK-4002, Vectastain, Vector Laboratories, CA, USA). As substrate and chromogen of peroxidase, peroxidase and 3,3′-diaminobenzidine (DAB) were used. Slides were counterstained with CAT hematoxylin. Molecules studied were quantified in 10 random fields at 40× magnification over the entire circumference of the sample using ImageJ software version 1.54 k (http://rsbweb.nih.gov/ij/) and the IHC Profiler plugin, which reports the number of positively stained pixels per case [50].

### 4.11. Biochemical Determination of Newly Formed Collagen (NFC) Deposition

NFC deposition in TS and tracheal scar tissue was quantified using the Sircol assay. Total protein concentration was measured by the Lowry method, and soluble collagen content was quantified using Sircol dye reagent (kit S1000, Biocolor, Northern Ireland, UK) following the manufacturer’s instructions. Absorbance was measured at 555 nm using an Epoch spectrophotometer (BioTek Instruments, Winooski, VT, USA) [10,40].

### 4.12. Statistical Analysis

Statistical analysis of clinical, macroscopic, and microscopic findings was performed using the Kruskal–Wallis and chi-square tests. Tomographic, biochemical, and immunohistochemical data were analysed using ANOVA, Dunnett, and Tukey tests. A *p*-value < 0.05 was considered statistically significant. All analyses were performed using SPSS version 26 for Windows (SPSS Inc., Chicago, IL, USA).

## 5. Conclusions

Based on our findings, the use of polymerised collagen or PFD as a stand alone treatment for 4 weeks does not reverse TS. However, it halts disease progression by increasing apoptosis, decreasing ITGβ-1 production, and reducing profibrotic growth factors and extracellular matrix proteins, as well as regulating MMP-1 and MMP-9 expression. Nevertheless, when used in combination with TRE, these agents promote favourable tracheal healing and prevent restenosis. These findings suggest that polymerised collagen and PFD may have potential as adjunctive therapeutic strategies for the management of tracheal stenosis and warrant further investigation in clinical studies, particularly in patients who are not suitable candidates for surgical intervention.

## Data Availability

The original contributions presented in this study are included in the article. Further inquiries can be directed to the corresponding author.

## Abbreviations

The following abbreviations are used in this manuscript:

Polymerised collagen	Polymerised type I collagen
PFD	Pirfenidone
TS	Tracheal stenosis
TRE	Tracheal resection
RTS	Tracheal restenosis
AB	Apoptotic bodies
ITGβ1	Integrin beta 1
TGF-β	Transforming growth factor beta
FN	Fibronectin
MMP	Matrix metalloproteinase
SS	Saline solution
MMC	Mitomycin C
ECM	Extracellular matrix
WHM	Wound-healing modulator
CT	Computed tomography
NFC	Newly formed collagen
TS-SS	Tracheal stenosis treated with physiological saline solution
TS-MMC	Tracheal stenosis with treated with extraluminal topical application of MMC
TS–polymerised collagen	Tracheal stenosis with polymerised collagen
TS-PFD	Tracheal stenosis treated with PFD
TRE-SS	Tracheal stenosis treated with TRE and physiological SS
TRE-MMC	Tracheal stenosis treated with TRE and extraluminal topical application of MMC
TRE–polymerised collagen	Tracheal stenosis treated with TRE and polymerised collagen
TRE-PFD	Tracheal stenosis treated with TRE and PFD
VEGF	Vascular endothelial growth factor
